# Seeking Optimal Region-Of-Interest (ROI) Single-Value Summary Measures for fMRI Studies in Imaging Genetics

**DOI:** 10.1371/journal.pone.0151391

**Published:** 2016-03-14

**Authors:** Yunxia Tong, Qiang Chen, Thomas E. Nichols, Roberta Rasetti, Joseph H. Callicott, Karen F. Berman, Daniel R. Weinberger, Venkata S. Mattay

**Affiliations:** 1 Clinical and Translational Neuroscience Branch, Division of Intramural Research Programs, National Institute of Mental Health, National Institutes of Health, Bethesda, Maryland, United States of America; 2 Lieber Institute for Brain Development, Johns Hopkins Medical Campus, Baltimore, Maryland, United States of America; 3 Department of Statistics, University of Warwick, Warwick, United Kingdom; 4 Department of Psychiatry, University of Pennsylvania, Philadelphia, Pennsylvania, United States of America; 5 Departments of Psychiatry, Neurology and Neuroscience, Johns Hopkins University School of Medicine, Baltimore, Maryland, United States of America; 6 Departments of Neurology and Radiology, Johns Hopkins University School of Medicine, Baltimore, Maryland, United States of America; Brown University, UNITED STATES

## Abstract

A data-driven hypothesis-free genome-wide association (GWA) approach in imaging genetics studies allows screening the entire genome to discover novel genes that modulate brain structure, chemistry, and function. However, a whole brain voxel-wise analysis approach in such genome-wide based imaging genetic studies can be computationally intense and also likely has low statistical power since a stringent multiple comparisons correction is needed for searching over the entire genome and brain. In imaging genetics with functional magnetic resonance imaging (fMRI) phenotypes, since many experimental paradigms activate focal regions that can be pre-specified based on *a priori* knowledge, reducing the voxel-wise search to single-value summary measures within *a priori* ROIs could prove efficient and promising. The goal of this investigation is to evaluate the sensitivity and reliability of different single-value ROI summary measures and provide guidance in future work. Four different fMRI databases were tested and comparisons across different groups (patients with schizophrenia, their siblings, vs. normal control subjects; across genotype groups) were conducted. Our results show that four of these measures, particularly those that represent values from the top most-activated voxels within an ROI are more powerful at reliably detecting group differences and generating greater effect sizes than the others.

## Introduction

The effect of genes on cognition and behavior is modulated through a complex series of intermediate steps including changes at the molecular level and alternations in neural circuits [1, 2]. In the past decade, imaging genetics has emerged as a promising genetic association analysis approach that uses a variety of state-of-the-art anatomical or functional brain imaging measures as phenotypes to evaluate the impact of genetic variation on the susceptibility to neuropsychiatric disorders [1, 3, 4]. The quantitative measures from so-called structural imaging (e.g., volume, cortical thickness, surface area, etc.) or brain function (physiological response of the brain during information processing or in the so-called “resting state”) derived from neuroimaging modalities are potential intermediate phenotypes that are both genetically associated with neuropsychiatric disorders and closely related to the underlying neural mechanisms of the disease process [4, 5].

Two general association-based approaches have been used in imaging genetics. The candidate gene association approach is a strategy for studying the effects of a well-defined candidate gene that is a good candidate because of its implication in the biological pathway of the disease on measures of brain biology and circuits. On the other hand, the genome-wide association (GWA) approach seeks to discover novel genetic loci which might be related to brain structure and function through screening the entire genome for potential associations. To date, a number of GWA studies of imaging phenotypes have been conducted and some of these studies reported multiple single nucleotide polymorphisms (SNPs) related to risk for schizophrenia and Alzheimer’s disease [6–10].

Imaging phenotype measures in GWAS depend on large groups of neurons whose structure and function are affected by multiple genes. While studying the relationship between multi-gene and multiple intermediate neuroimaging phenotypes can generate exciting new discoveries, it places a big challenge for researchers to choose an overall valid strategy and apply suitable computational tools and statistical methods [11, 12]. There are two major issues related to the GWAS approach in imaging genetics. One is that relating whole genome and whole brain data requires immense computational power [13]. Therefore, prior studies have typically made a significant reduction in imaging space and only examined a few imaging variables, such as using magnetic resonance imaging (MRI) measures of hippocampal atrophy [14], total cerebral brain volume (TCBV), white matter hyperintensity (WMH) volumes, etc. [15]. Secondly a whole brain voxel-wise approach in genome-wide searches would also likely have low power. A stringent multiple comparisons correction is needed for searching over the entire genome and all the voxels in the brain to control for the number of false positives.

Resolving this issue is especially critical for studies using functional magnetic resonance imaging (fMRI), a popular neuroimaging tool used in Imaging Genetics studies. A practical solution is to apply dimensionality reduction on the acquired fMRI time series that contains tens of thousands of voxels in a single brain volume.

Traditionally, the first step toward dimensionality reduction of fMRI data is built upon model-driven approaches such as performing a general linear model to reduce the 4-D time series to a 3-D statistical parametric map (SPM) for each subject. And then a second-level voxel-wise statistics is performed to find activated voxels across subjects [16]. However, correction for multiple comparisons across all the voxels results in much reduced statistical power [17]. In addition, due to the anatomical and functional variability between brains of different individuals, the peak locations of the regional activation vary significantly across subjects, even with careful spatial normalization and smoothing [16]. Therefore, the sensitivity of the group-level analysis may be compromised by this approach [18].

Because many experimental paradigms in imaging genetics activate focal regions that can be pre-specified based on a priori knowledge, reducing the whole-brain search to single-value summary measure of ROIs is a viable approach, as evaluated in the current study. Two questions arise when performing the ROI analysis. The first question is how to select an appropriate ROI [19, 20] for extracting the single-value summary measure. The second, potentially more difficult question, is how to find an ROI measure that is robust and sensitive to group differences [21], which is the primary focus of the current study.

In order to investigate the effects of different ROI summary measures on the group analysis of fMRI activation, ten ROI summary measures were chosen for several considerations. The most commonly used ROI summary measures are to simply take the ROI mean or median. However, due to volume averaging artifacts in fMRI, the mean or median summary over a multi-voxel ROI may not optimally represent activity within the ROI as one region may not be functionally homogenous, that is, activated voxels may be grouped with inactive or de-activated voxels resulting in lower summary measures [18, 21]. One way to reduce the influence of these voxels of no interest is to use the first eigenimage [22] to compute a weighted ROI mean. Another strategy is to select voxels above a certain threshold, i.e., “activated” or “top fraction” voxels in an ROI [20]. In this study we used either the individual voxel’s *p* values (*p* = 0.05) or percentile (*q* = 25^th^ %, 10^th^ %, or 5^th^ %tile of all voxels) as the threshold. These above-threshold voxels are regarded as showing significant difference in BOLD response across task sessions underlying the cognitive/behavioral process being tested and are more likely to represent true activity within the ROI.

Another set of activation-based ROI summary measures is to select voxels surrounding each individual’s ROI maxima (peak) of activation [21]. Choosing the active voxels around individual peaks can accommodate the inter-subject variability in the location of activation [23]. Due to activation fluctuation, a single peak voxel could contain extreme value that may not best represent the individual’s activation in the ROI. Selecting a group of activated voxels surrounding the peak and using the mean as the ROI single-value summary measure can help reduce the between-subject variance. Here we used several strategies to select voxels surrounding peak voxels: top N (N = 10, 20, 50) contiguous voxels surrounding the peak, a sphere surrounding the peak, an activated cluster surrounding the peak, or voxels within the ROI whose time courses are highly correlated with the peak voxel’s time course. Finally, we also examined the number of activated voxels (i.e., the *extent* measure), which is a commonly used ROI summary measure in fMRI studies [24].

While the ROI approach can increase statistical power, strong prior hypotheses of the particular ROI used are required. These prior hypotheses of ROI selection can either be based on published literature of the task of interest [21], made on an anatomical basis, or focused on the functional activation induced by the task [25]. Despite of more advanced ROI selection methodologies based on multivariate pattern analysis (e.g., independent component analysis, ICA) [19], since the primary goal of this work was to assess and compare the ten ROI-based single summary measures described above, we chose the three simple, univariate ROI definition strategies to detect group differences in an attempt to provide practical guidance for future work for selecting the most optimal imaging phenotype/measure in large scale genome-wide imaging genetics studies.

The number of voxels in an ROI may also affect the effect size. In general, we expect to see greater variability between ROI summary measures in ROIs that contain a larger number of voxels such as the anatomical ROIs. As for the smaller 10-mm spherical ROI, the voxels selected using the different ROI summary measures may largely overlap and result in similar effect sizes across the measures.

In this project we examined the ROI single-value summary measures using four datasets, with the purpose to test the reliability of each measure across datasets. Firstly, it has been consistently observed that patients with schizophrenia (PTs) show increased brain activity in DLPFC regions compared with normal control subjects (NCs) during the NBack working memory task when performance does not differ between the groups [26]. We compared the replicability of the ROI summary measures by comparing two performance matched groups (PTs vs. NCs) using an NBack task dataset that was split into two matched subgroups.

The unaffected siblings (SIBs) of patients with schizophrenia have also been shown to manifest DLPFC hyperactivity compared with control subjects with similar levels of performance [27], suggesting this phenomenon to be a heritable trait measure related to genetic risk for schizophrenia. Therefore we included unaffected siblings in the second dataset to examine which of the ROI summary measures tested are more sensitive to uncover genetic risk shared by patients with schizophrenia and their unaffected siblings.

The third dataset was chosen to test the validity of the different ROI summary measures in an imaging genetics study. This is a particularly relevant investigation, since genetic variants have typically been shown to have very subtle effects on imaging measures (less than 1% per allele), which is much lower than the effect *due to any disease process that is discernable with neuroimaging* [28]. For this part of the study, we chose a dataset comprised of NBack fMRI data in healthy volunteers from three genotype groups related to a common functional polymorphism (Val^108/158^ Met) in the Catechol-O-methyltransferase (COMT), which accounts for variation in enzymatic activity and cortical dopamine catabolism. This was based on prior evidence that the Val^158^ allele of COMT is associated with inefficiency in prefrontal cortex information processing and lower performance during the NBack working memory task in an allele dosage fashion Val/Val > Val/Met > Met/Met [29]. Therefore, we expect to observe greatest DLPFC activation during the N-Back working memory task in the Val/Val group when compared to the Val/Met group who would show greater activation than the Met/Met group. Despite the differences in subjects populations, the above three datasets used the same blocked design NBack paradigm. To test if the results from the analyses of N-Back block design data could be generalizable to event-related data for a different cognitive process and ROI, we examined the ROI summary measures using an event-related modified Flanker task dataset. In a previous study [30], patients with schizophrenia and their unaffected siblings showed decreased dorsal anterior cingulate cortex activation relative to normal controls during No-Go condition.

## Methods

### Participants

There were three groups of subjects whose data were used in this project: patients with schizophrenia (PTs), their unaffected siblings (SIBs), and normal control subjects (NCs). All participants were recruited nationwide as part of an ongoing family study of schizophrenia at the Clinical Brain Disorders Branch, NIMH, NIH (Protocol 95-M-0150, Dr. Weinberger PI). The study was approved by the institutional review board of the Intramural Program of the National Institute of Mental Health, National Institutes of Health. All participants were assessed with a Structured Clinical Interview for DSM-IV (APA, 1994). They were prescreened to exclude those with premorbid IQ below 70, those with recent drug or alcohol abuse (within 1 year) or more than 5 years of previous abuse, and those with medical or neurological conditions. Most PTs were on a stable regimen of antipsychotic medication (typical neuroleptics and atypical antipsychotics). SIBs and NCs were selected with the additional requirements that they are not on any current psychotropic pharmacological treatment. Further, NCs should not have a first-degree relative with a schizophrenia spectrum disorder. After a complete description of the study to the subjects, written informed consent was obtained.

### Experimental Procedures and Image Acquisitions

The datasets used in current study were collected during two cognitive tasks: the NBack task [27] and the modified Flanker task [31].

#### NBack Task

The NBack working memory task consisted of four 30-sec blocks of the 0-Back condition alternating with four blocks of the 2-Back condition, in which a random series of numbers (1 to 4) were presented every 2 seconds for 500 milliseconds at set locations in a diamond-shaped box. During the 0-Back condition, participants were asked to respond to current number being presented by pressing the corresponding keys on a keypad; in the 2-Back condition, participants encoded the number currently being shown and simultaneously recalled and responded to the number presented two stimuli earlier.

BOLD fMRI was performed on a General Electric (GE) Signa 3T scanner (GE Healthcare, Waukesha, WI, USA). A gradient-echo EPI (Echo Planar Imaging) sequence was used to acquire 120 images (24 interleaved 6 mm axial slices, in-plane resolution = 3.75 × 3.75 mm^2^, FOV = 24 cm^2^, matrix = 64 × 64, TR/TE = 2000/28 ms, flip angle = 90°).

Images were preprocessed in SPM5. The first four scans were discarded to allow for signal saturation. Images were realigned to the first image of the scan run, spatially normalized into a standard stereotactic space with a voxel size of 3 mm isotropic [Montreal Neurological Institute (MNI) template] by using affine and nonlinear transformations, smoothed with a full-width half-maximum (FWHM) Gaussian filter (8 mm) and ratio normalized to the whole-brain global mean. All fMRI data were individually examined for motion artifacts and excluded for excessive intra-scan motion (>2 mm translation, >1.5° rotation). A statistical image for the contrast of 2-Back Vs. 0-Back was then obtained for each subject.

The effect of interest was the 2-Back versus 0-Back contrast, using which abnormal right dorsolateral prefrontal cortex (rDLPFC) function had been previously reported in patients with schizophrenia and unaffected siblings [27].

#### Flanker Task

The modified Flanker task [31] is an event-related task designed to evaluate response inhibition and interference suppression. Each session consisted of four experimental conditions and a total of 145 trials. Each trial was presented for 800 ms that included five symbols with the central arrow pointing left or right, flanked by four symbols, two on each side. In three conditions (‘congruent’, ‘incongruent’, ‘neutral’), the subjects were asked to indicate the direction of the central arrow on the screen while ignoring the four flankers. During the ‘No-Go’ condition, the subjects were instructed to withhold their motor response when the flankers were ‘X’s. In current project, we limited our analysis to the correct trials in the No-Go condition to focus on response inhibition process.

BOLD fMRI was performed on a GE Signa 3T scanner. A gradient EPI sequence was used to acquire 300 images (26 axial slices, 4 mm thickness with 1 mm gap, in-plane resolution = 3.75 × 3.75 mm^2^, FOV = 24 cm^2^, matrix = 64 × 64, TR/TE = 2000/28 ms, flip angle = 90°).

Images were preprocessed following the same procedures as NBack task (slice-timing correction, motion correction, spatial normalization and smoothing) in SPM5. A contrast image for response inhibition (No-Go) was generated per subject per session using the correct trials only.

### Datasets

As described earlier, we tested the ROI single-value summary measures using four datasets: 1) the NBack task split dataset, 2) the NBack task sibling dataset, 3) the NBack task COMT dataset, and 4) the Flanker task dataset.

#### The NBack task split dataset

This dataset consisted of 100 PTs and 100 NCs. To compare the replicability of the ROI summary measures, the dataset was split into two equal subsets. Each subset comprised of 50 PTs and 50 NCs. The NCs in each of the split subsets were matched (*t* test *p* values were all above 0.2; see S1 Table) for age, gender, premorbid IQ estimated by the scores on wide range achievement test (WRAT), handedness, task performance on 2-Back blocks (% correct), and the image time course stability as measured by temporal signal-to-noise ratio (TSNR), which is calculated by dividing the mean of the time course by its standard deviation (TSNR; [32]). The two NC groups and the two PT groups were also matched, respectively.

In addition, to validate the robustness of our results and to ensure good replication power, we ran many random samplings (without replacement) on this dataset. That is, from among the 100 PTs in the first dataset, we used an automated script to randomly choose 50 of them during each iteration. Then among the 100 NCs in the same dataset, we selected 50 NCs that were matched for age, gender, WRAT, and 2-Back accuracy (*t* test *p* values were all above 0.2) with the 50 PTs. We repeated this random sampling and matching procedure 1000 times so that each sampled subset contained 50 PTs and 50 matched NCs that were matched for age, gender, WRAT, and 2-Back accuracy.

#### The NBack Sibling dataset

The dataset include three groups: NCs, SIB, and PTs, with 43 subjects in each group. To reduce the confound effect of task performance on the prefrontal activation [33], we only selected the subjects with relative high performance in the 2-Back task with the percentage of accuracy greater than 70%. The three groups were also matched for age, gender, WRAT score, handedness, percent correct on the 2-Back WM task, and TSNR (one-way ANOVA *p* values were all above 0.2; see S2 Table).

#### The NBack COMT dataset

A third dataset consisted of NBack data from 216 NCs (43 Val/Val individuals, 106 Val/Met individuals, and 67 Met/Met individuals). The three groups were matched for age, gender, WRAT score, handedness, percent correct in 2-Back task, and TSNR (one-way ANOVA *p* values were all above 0.2; see S3 Table).

#### The Flanker task dataset

Our current dataset include two groups: 28 NCs and 28 PTs, which is a subset of the data used by Sambataro et al. [30] with the two groups matched for age, gender, WRAT score, handedness, percent correct on No-Go task, and TSNR (*t* test *p* values were all above 0.2; see S4 Table).

### Data Analysis

For each of the datasets, three types of ROI definition strategies were used for extracting the single summary values: a smaller spherical ROI based on published literature (see details below), a bigger anatomical ROI based on an atlas, and a task-activated cluster constrained by the anatomical ROI. See S1 Fig.

#### The ROI masks (S1 Fig)

For the NBack task dataset 1 and 2, the three types of ROI masks were: 1) A 10-mm sphere ROI placed in the right dorsolateral prefrontal cortex (rDLPFC) and centered on the coordinates MNI xyz = [40 31 34] obtained from a published meta-analysis (Owen et al., 2005); 2) Right DLPFC anatomical ROI. The ROI was created by combining the right BA46 and lateral BA9 and dilated by 2 voxels from the Brodmann Area Atlas provided by Wake Forest University PickAtlas (WFU PickAtlas; www.fmri.wfubmc.edu/downloads), with the medial aspect removed; 3) Task-activated cluster in the right DLPFC, which was defined using the following steps: For each of the NC and PT groups, a main effect of task map was created using a one-sample *t* test across all the subjects in that group. Then for each of these group maps, the voxels above *p* < 0.05 familywise error (FWE) correction within the right DLPFC anatomical mask were deemed as task-activated voxels. A joint map between the two group maps was used as the ROI mask.

The ROIs used for the NBack task dataset 3 were the same as those used for dataset 1 and 2 except for the center of 10-mm spherical ROI based on previous literature. Egan et al. [29] reported a greater activation in right DLPFC for Val/Val > Val/Met > Met/Met contrast with the peak located in BA 46 (MNI xyz = 58 32 12) during 2-Back task. Therefore, we chose this peak location as the center for the 10-mm spherical ROI, with x coordinate shifted to 52 so that the voxels in this 10-mm radius ROI are within the brain.

For the Flanker task dataset 4, three ROIs were used 1) A 10-mm sphere ROI placed in the dorsal anterior cingulate (ACC) centered on MNI coordinates xyz [0 15 36] obtained from Sambataro et al. [30]; 2) Anatomical ACC ROI by combining BA 24 and BA 32 and dilated by 1 voxel from the WFU PickAtlas; 3) Similar to the NBack task ROI mask, a joint map in an anatomical ROI encompassing ACC based on activations in NC and PT group maps was used as the ROI mask.

#### ROI Single Value Summary Measures

We used ten different ROI summary measures to extract single-value summaries from an ROI, see Table 1. The procedures of voxel selections for each ROI summary measure are described below. For measures 3 to 10, we also masked the ROI with a threshold (*p* = 0.05) to select the activated voxels.

**Table 1:** The methods for extracting values from an ROI.

	A: Without applying p threshold	B: Only include voxels above p threshold of 0.05
**1**	Mean	Mean
**2**	Median	Median
**3**	ROI mean weighted by eigenimage	ROI mean weighted by eigenimage
**4**	Voxels above percentile (75%tile, 90%tile, or 95%tile) threshold	Voxels above percentile (75%tile, 90%tile, or 95%tile) threshold
**5**	ROI peak	
**6**	Top 10, 20, or 50 voxels around the ROI peak	
**7**	Voxels within a sphere (radius = 6, 7.5, or 10 mm) around the ROI peak	Voxels within a sphere (radius = 6, 7.5, or 10 mm) around the ROI peak
**8**	Voxels whose timecourse are highly correlated with the peak voxel	Voxels whose timecourse are highly correlated (r = 0.7, 0.8, 0.9) with the peak voxel
**9**		Voxels in cluster containing ROI peak
**10**		Extent of ROI peak cluster

All ROI summary values were extracted individually from each subject’s first level contrast map (e.g., 2Back > 0Back contrast) to account for between-subject variation in functional activation patterns. All thresholds are set at an individual subject level.

1. Mean
  - Mean linear fit coefficient of all voxels in the ROI.
  - The mean linear fit coefficient of the voxels above a *p* threshold (*p* = 0.05, uncorrected in this study) in the ROI.
2. Median
  - Median linear fit coefficient of all voxels in the ROI.
  - The median linear fit coefficient of the voxels above a *p* threshold (*p* = 0.05) in the ROI.
3. Contrast map mean weighted by the first eigenimage of an ROI
  Eigenimages of an ROI were obtained using singular value decomposition (SVD), which is an operation that decomposes an original time-series (M) from all voxels in the ROI into two sets of orthogonal vectors V (patterns in space) and U (patterns in time) where:
  [U,S,V]=SVD(M)
  Here S is a diagonal matrix of decreasing singular values [22]. The first eigenimage is the pattern that accounts for the greatest amount of the variance-covariance structure. We use it to weight the contrast map so that the voxels that represent the most common pattern of the activation in an ROI are enhanced and the other irrelevant voxels are suppressed.
  - For each individual subject’s time series, we extracted the timecourses of all the voxels within the ROI and then the first eigenimage of these voxels was calculated by SVD. Each voxel’s linear fit coefficient was weighted by this eigenimage and the weighted mean was taken as the ROI summary measurement.
  - Instead of using all voxels in the ROI, we first selected only the voxels above a *p* threshold (*p* = 0.05). Then the first eigenimage of the selected voxels was calculated and used to weight the linear fit coefficient of each above-threshold voxel. The weighted mean was taken as the ROI summary measurement.
4. Voxels above percentile threshold
  - We selected voxels whose t values were among top percentiles (25^th^ %tile, 10^th^ %tile, or 5^th^ %tile) of all voxels. The mean of the linear fit coefficient of these voxels was taken as the ROI summary measurement.
  - We first selected the voxels above a *p* threshold (*p* = 0.05) in the ROI. Then a percentile threshold was applied to these the above-p-threshold voxels. The mean of the linear fit coefficient of these selected voxels was used as the ROI summary measurement.
5. Peak voxel within the ROI
  For this approach, we chose the voxel with the maximum *t* statistic in an ROI from the SPM T map, and then from the SPM contrast map we extract this voxel’s linear fit coefficient (beta), which is essentially the slope value from the first-level multiple regression, as the ROI’s summary measurement.
6. Top N contiguous voxels surrounding the peak
  Because BOLD fMRI is affected by various types of noise, using only the peak voxel value may bias the estimation of an ROI. Starting from the peak, this “top-down” voxel search is to find a group of contiguous voxels with highest *t* values surrounding the peak. Similar to a breadth-first search, the algorithm grew the voxel set by first visiting the 26 voxels adjacent to the peak, and then sorting the voxels by their *t* values to ensure that the obtained set contain the highest activated voxels. The search stopped when the number of voxels N reached a certain threshold (N = 10, 20, 50 voxels). If N did not reach the threshold after the first round (in this case, 50), then in the second round, the search visited the voxels surrounding the top voxels in the sorted set, and so on. After the search, these voxels’ mean linear fit coefficient was used as the ROI summary measurement.
7. Voxels within a sphere surrounding the peak
  - We selected the voxels within a sphere (radius = 6, 7.5, or 10 mm) surrounding the peak of the *t* contrast map. Then the mean of the linear fit coefficient of these within-sphere voxels was taken as the ROI summary measurement.
  - First the voxels above a *p* threshold (*p* = 0.05) in the ROI were extracted. Then the above-threshold voxels within a sphere (radius = 6, 7.5, or 10 mm) surrounding the peak were picked and the mean linear fit coefficient of these voxels was taken as the ROI summary measurement.
8. Voxels’ timecourses correlated with the peak voxel
  - We computed the correlation coefficients between the peak voxel’s timecourse and the timecourses of all other voxels in the ROI. We used different correlation coefficient thresholds (r = 0.7, 0.8, and 0.9) to select the voxels that are highly correlated and clustered together with the peak. The mean of the linear fit coefficient of these selected voxels was used as the ROI summary measurement.
  - We first selected voxels that are above a *p* threshold (*p* = 0.05) in the ROI. Then a similar algorithm as in 8A was applied to find the voxels that are highly correlated with the peak voxel.
9. Cluster containing ROI peak
  We used a *p* threshold (*p* = 0.05) to extract the voxels that are considered activated in the task. These voxels may form several clusters in the ROI. We selected only the voxels in the same cluster as the peak and then computed the mean linear fit coefficient of these voxels. There was no restriction for the maximum cluster size. If there were no voxels above the *p* threshold, then the individual’s data was considered missing in the group analysis.
10. Extent of ROI peak cluster
  We applied a *p* threshold (*p* = 0.05) and identified clusters of activated voxels from the above-threshold voxels. The number of voxels within the cluster that contained the peak was used as the ROI summary measurement.

#### Statistical Analysis

All extracted data were analyzed in Statistical Analysis System (SAS) software developed by SAS Institute Inc., Cary, NC. Two-sample *t*-tests were performed to test these measures’ ability to detect a significant difference in activation between schizophrenia patients and normal controls for dataset 1 and 4. One-way ANOVAs were used to test the sensitivities of different ROI summary measures in finding the significant group effect, i.e., PTs > SIBs > NCs for dataset 2 and Val/Val > Val/Met > Met/Met for dataset 3.

We used the effect size to gauge the power of a test. Cohen’s *d* was used to explore the effect size index for the *t* tests [34], and an Omega Squared test was performed to test the effect size index for the ANOVAs [35, 36]. The power of a test is monotonically related to its effect size, and a test with a larger effect size will have better power (for a given sample size).

#### Test of Small ROI Localization Differences between Groups

Any differential effects observed across the different summary measures may have been influenced by whether all the voxels in an ROI or only a subset of voxels within an ROI are chosen to create the summary measure. Here we tested whether the localization of the selected cluster of voxels is different across the groups in our study. Since many of the ROI summary measures were related to selecting voxels surrounding each individual subject’s peak, as a result, a between-group difference derived from such a measure may be driven by a difference in peak locations between groups. This issue was further examined by a permutation test.

We first extracted the peak coordinates within the anatomical ROIs from each individual subject’s contrast map. Next, the Euclidean distance between the centroids of peak coordinates among all the subjects in each group was computed (for three or more groups, the maximum distance between each pair of centroids was used). Then we randomly assigned the group label of each subject and computed the Euclidean distance between the centroids of these randomly formed groups (each group contained exactly the same number of subjects as the original dataset). The permutation test on this Euclidean distance was repeated for 1000 times. We then computed the percentile of the actual group centroid distance against the distribution of random distance obtained from the permutation test. The hypothesis is that if the peak locations are different across the groups, then the actual group centroid distance will have a much higher percentile (>95%) in the distribution.

## Results and Discussion

The aim of the present work was to compare the effect of ten different implementations of ROI single value summary measures on the results of group analysis, including both diagnostic groups (patients with schizophrenia vs. normal control subjects) and genetic groups (COMT Val & Met polymorphism, Val/Val, Val/Met, Met/Met). We also compared the three different types of ROI selection strategies generally adapted by researchers in the literature: 1) a spherical ROI centered around previously published group peak coordinates; 2) an anatomical ROI encompassing multiple Brodmann areas based on *a priori* information; and 3) task-activated cluster within an anatomical ROI. We found that four ROI single-value summary measures were reliably consistent across datasets to show significant group differences and produce relatively higher effect sizes: 1) top percentile (measure 4), 2) the top N contiguous voxels surrounding the peak voxel (measure 6); 3) a sphere surrounding the peak voxel (measure 7); and 4) peak-correlated voxels (measure 8). Different ROI summary measures are more similar when using the smaller, focal spherical ROIs.

### ROI Summary Measures versus Voxel-wise SPM Analysis

We first tested the replicability of these ROI summary measures in the split dataset, in which 100 NCs and 100 PTs were split into two subsets. The voxel-wise analysis showed a significant difference between PTs and NCs, using small volume correction within the anatomical DLPFC ROI implemented in SPM8 when data from all the PTs and NCs were included [37]. However, for each subset, the significance of group differences did not survive correction (*p >* 0.001 uncorrected for both subset 1 and 2; see Table 2).

**Table 2:** Group difference results from the voxel-wise analysis within anatomical ROIs.

Dataset	x	y	z	Z	PFWE-corr
**Dataset 1: 2 Back > 0 Back contrast, PTs vs. NCs in right DLPFC anatomical ROI**					
**All subjects: 100 PTs vs. 100 NCs**	36	30	36	3.62	0.04
	60	9	27	3.47	0.07
**Subset 1: 50 PTs vs. 50 NCs**	39	30	33	3.01	0.24
**Subset 2: 50 PTs vs. 50 NCs**	33	27	39	3.11	0.19
**Dataset 2: 2 Back > 0 Back contrast, 43 PTs vs. 43 SIBs vs. 43 NCs in right DLPFC anatomical ROI**					
	30	33	39	3.29	0.13
**Dataset 3: 2 Back > 0 Back contrast, 43 Val/Val vs. 106 Val/Met vs. 67 Met/Met in right DLPFC anatomical ROI**					
	51	39	9	4.16	0.01
**Dataset 4: Flanker NoGo contrast, 28 NCs vs. 28 PTs in Anterior Cingulate anatomical ROI**					
	9	21	36	4.03	0.03

**Fig 1:**
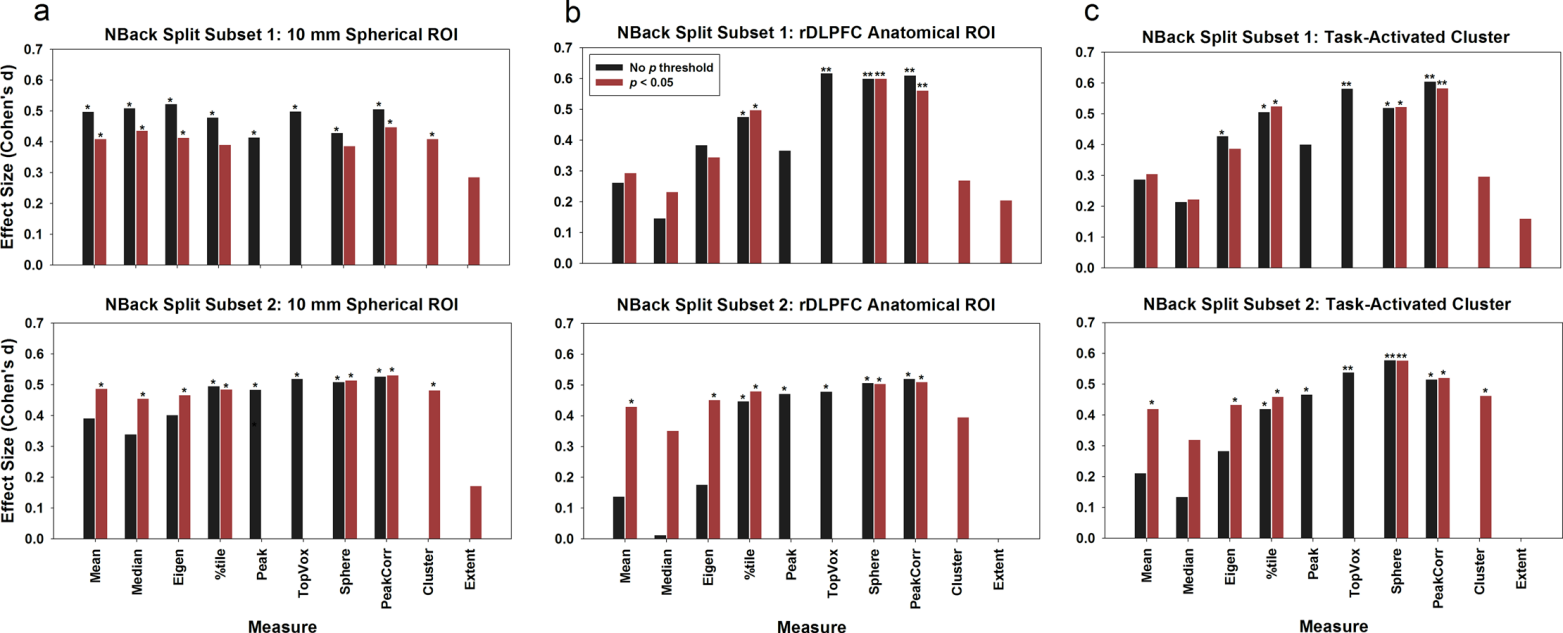
Using the NBack split dataset to evaluate the replicability of ROI summary measures. Three different strategies for ROI selection were used: (a) a 10-mm spherical ROI centered around the MNI coordinates xyz = [40 31 34]; (b) an anatomical ROI encompassing BA46 and lateral BA9; and (c) a task-activated cluster ROI. Asterisks denote significance level in group comparisons: * *p* < 0.05; ** *p* < 0.01.

In comparison, for the ROI summary measurements, except for measure 10 (number of activated voxels), 9 out of the 10 measures revealed a significant difference between the NCs and PTs for the 10-mm spherical ROI (*p* < 0.05), and the results were agreeable in both subsets (see Fig 1); measures 4, 6, 7, and 8 revealed significant group differences for the anatomical ROI (*p* < 0.05) in both subsets; measures 3, 4, 6, 7, and 8 revealed significant group differences for the task-activated ROI (*p* < 0.05) in both subsets.

**Fig 2:**
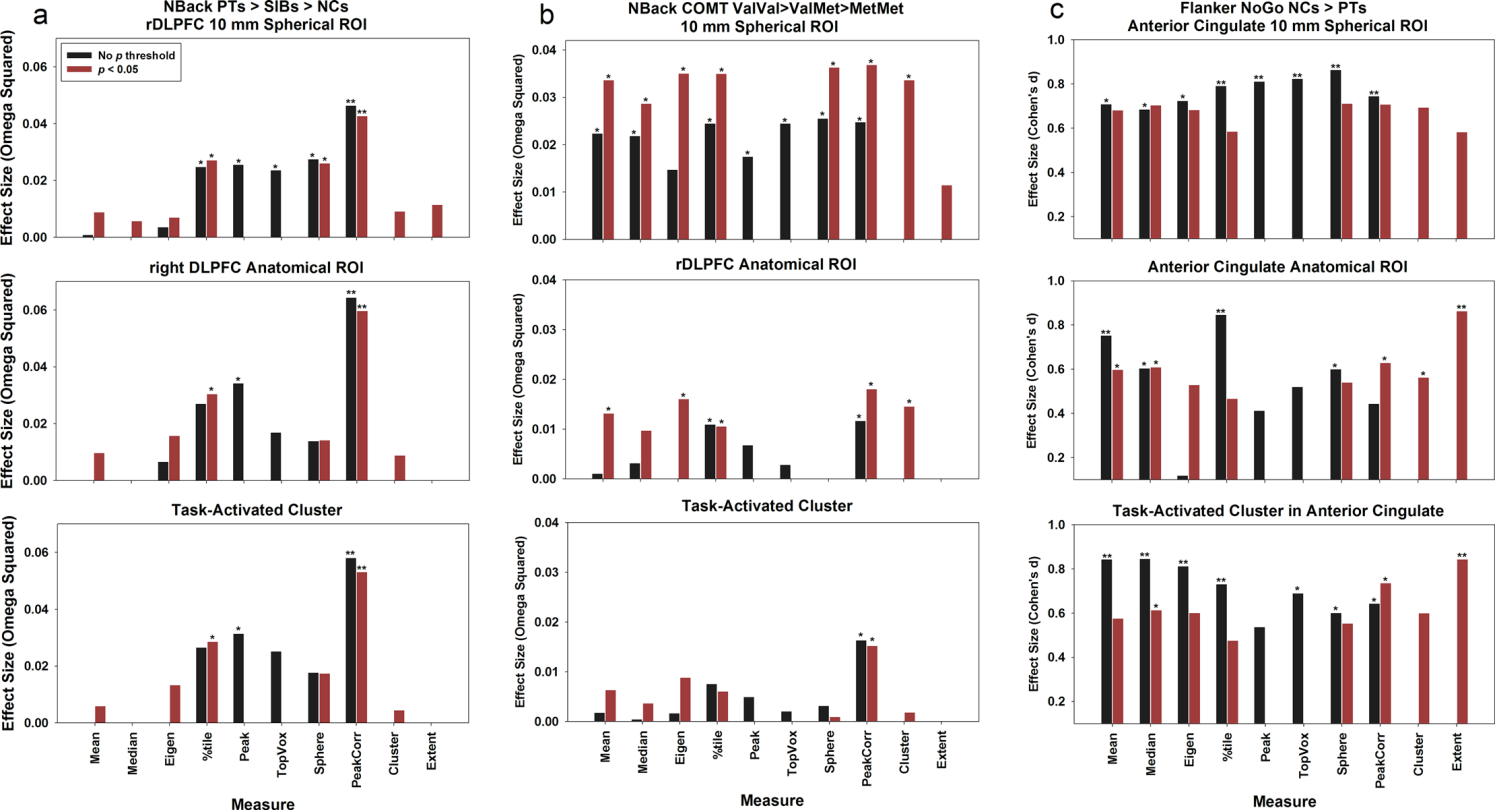
The generalizability of the ROI single value summary measures is further examined across three datasets. (a) The NBack Sibling dataset, PTs vs. SIBs vs. NCs, one-way ANOVA; (b) the NBack COMT dataset, Val/Val vs. Val/Met vs. Met/Met, one-way ANOVA; and (c) the Flanker dataset, NCs vs. PTs, *t* test.

We extended the comparisons to datasets 2, 3, and 4 (Table 2 and Fig 2). For the NBack Sibling dataset, we observed a trend but no significant difference for group comparison PTs > SIBs > NCs (*p* = 0.13 FWE-corrected). However, significant differences were found for measures 4, 5, and 8 (*p* < 0.05) across the three types of ROIs. For the NBack COMT dataset and the Flanker dataset, significant group differences were observed in both the voxel-wise analysis and the ROI summary measures (*p* < 0.05).

In summary, the ROI summary measures always demonstrated a group difference whereas in half of the datasets the voxel-wise approach sometimes failed to show a significant effect. One advantage of extracting ROI summary over the voxel-level analysis is that the ROI summary is extracted at the individual subject level, partially compensating for inter-subject anatomical and functional variability. Even though the image data have been normalized to standard space and spatially smoothed, there is still a significant degree of variability across subject’s anatomy [23]. In addition, even though the voxels across subjects can be aligned perfectly, the activation may not be. By expanding the search domain to the whole ROI and extracting individual-specific peak activation, it is more likely to precisely pinpoint the real activity for each individual.

### ROI Summary Measures and Types of ROI

**Fig 3:**
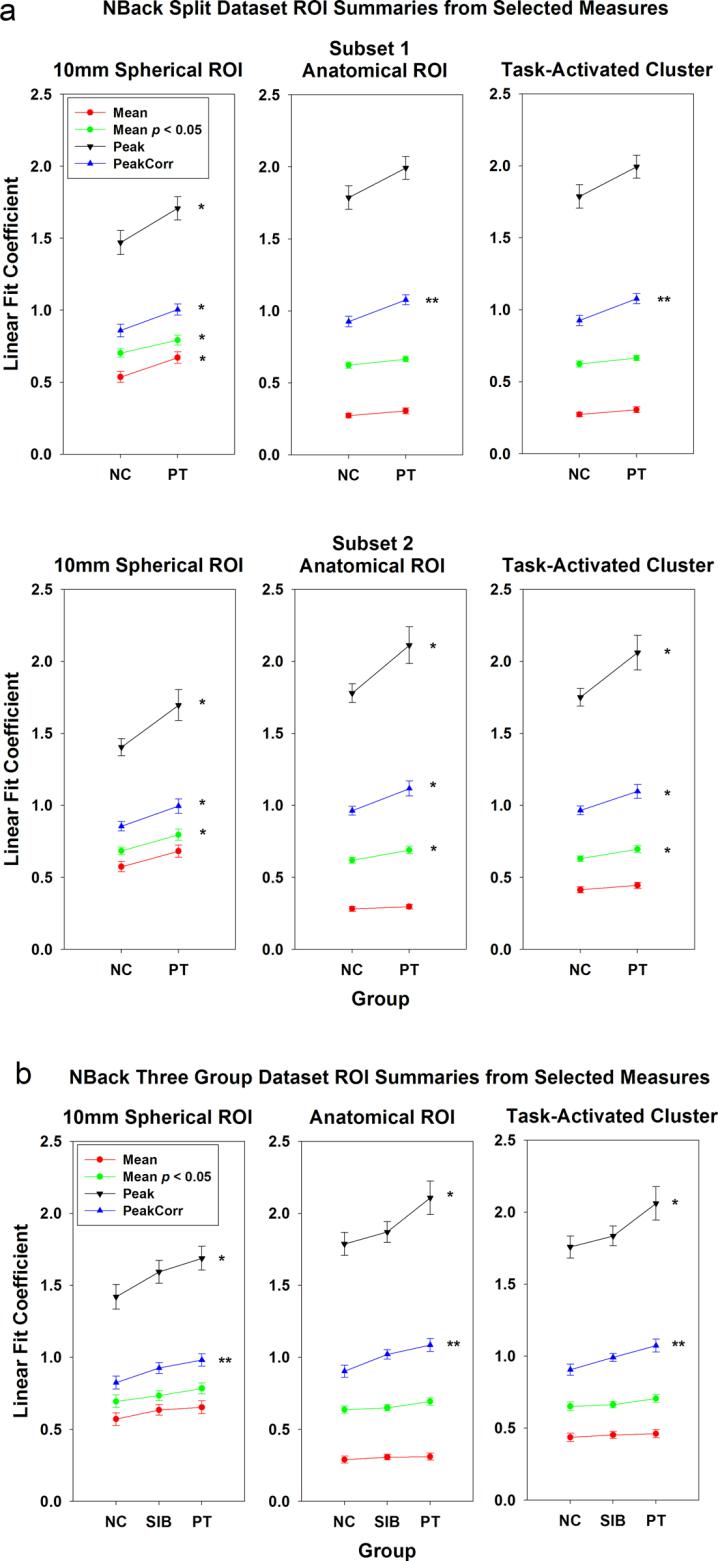
Group mean plots for two NBack datasets. (a) The split dataset PTs vs. NCs; and (b) the Sibling dataset, PTs vs. SIBs vs. NCs. Selected ROI summary measures (mean, mean of voxels above *p* = 0.05 threshold, peak, and peak-correlated voxels) were used to extract single-value summaries from three types of ROIs in right DLPFC. * *p* < 0.05; ** *p* < 0.01. Error bars represent standard errors.

Three types of ROIs were used for single-value extraction: 10-mm sphere, anatomical ROI, and task-activated cluster. Overall, as depicted in Figs 1 and 2, more ROI summary measures that generated significant results (*p* < 0.05) in the 10-mm spherical ROI than for the other two ROI approaches. For the first dataset, 9 ROI summary measures for the 10-mm spherical ROI showed significant group differences as compared to 4 ROI summary measures for the anatomical ROI and 5 ROI summary measures for the task-activated ROI (Fig 1); for the second dataset, the numbers are 5 (10-mm spherical ROI), 3 (anatomical ROI), and 3 (task-activated ROI) (Fig 2, panel a); for the third dataset, the numbers are 9, 5, 1 (Fig 2, panel b); and for the fourth dataset, the numbers are 8, 7, 8 (Fig 2, panel c). One explanation is that the 10-mm spherical ROI was created based on a known focal location that was active during the task and significant group differences had been observed previously. Therefore, the mean of the 10-mm spherical ROI was greater than those of the other two ROIs (*p* < 0.001; Fig 3, S2 Fig, red lines). For this approach, a strong *a priori* hypothesis is required to create such an ROI, i.e., one needs a meta-analysis or a previously published study that has shown significant group difference in this region to locate the center of the ROI, which could vary based on the comparison group being tested. For example, the ROI centered at BA9 that was used in dataset 1 and 2 could not apply for dataset 3 because a significant group difference for COMT genotype effect was observed at a different location in BA46 by Egan et al. (2001).

As shown in Fig 3 and S2 Fig, the peak measures were greater for the anatomical and task-activated ROIs (*p* < 0.001). Further analysis showed that for dataset 1, only 17.5% of individual subjects’ peak locations in right DLPFC were within the limited 10-mm spherical region. This indicates that due to individual’s anatomical and functional variation in peak activity, the small spherical ROI around MNI coordinates chosen from published literature cannot include all subjects’ peak activity locales.

### ROI Summary Measures Comparisons

The most important question we attempted to answer in this study is which ROI summary measures can consistently generate a high effect size and significant group differences that are reproducible. We first start the comparisons between with and without individual voxel p value thresholds, and then compare these measures across datasets.

#### The effect of applying individual voxel p value threshold

**Fig 4:**
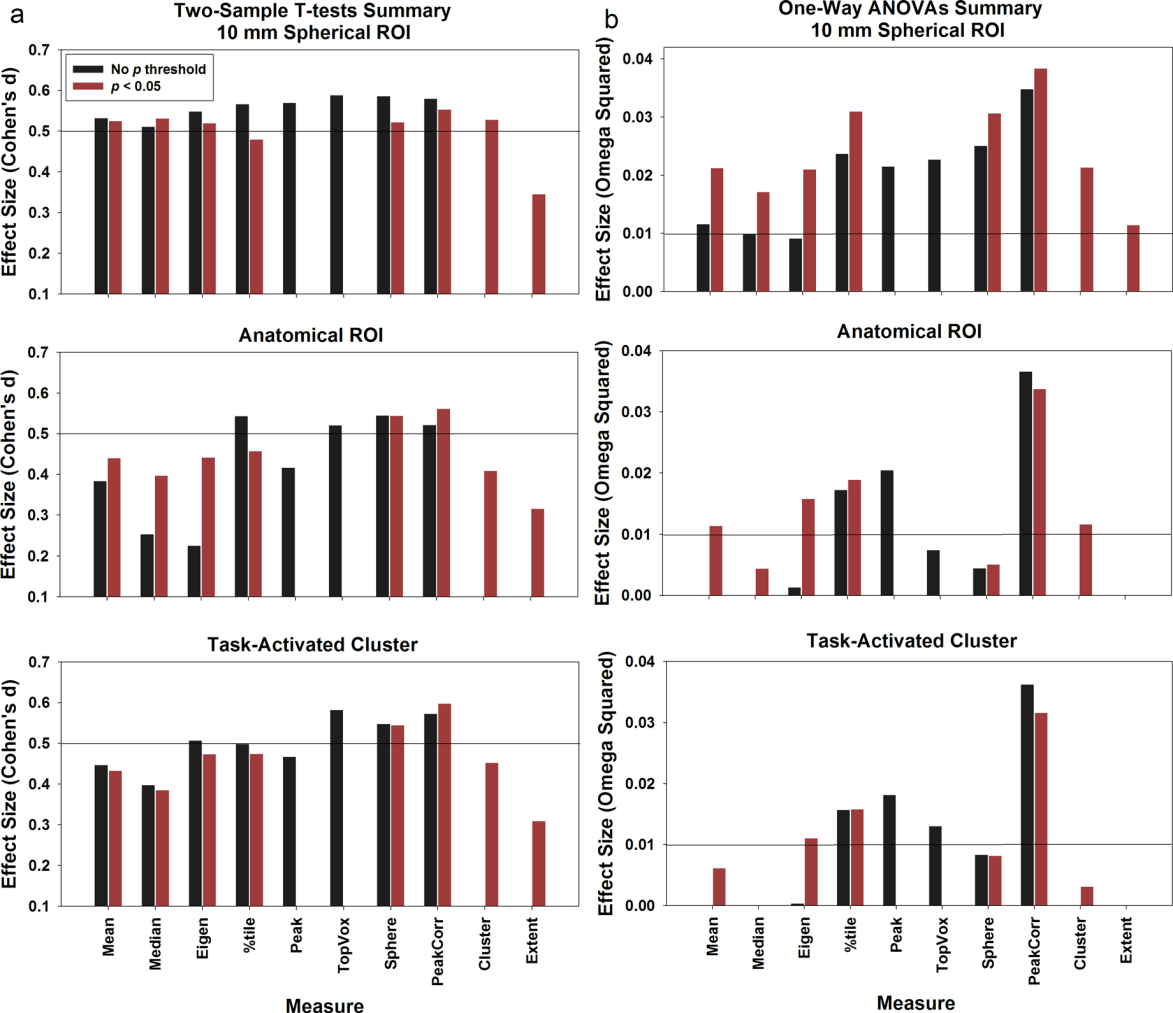
Results summary. We (a) combined the results of the two-sample *t* tests by averaging the effect sizes from dataset 1 and 4 together; and (b) combined the one-way ANOVA results by averaging the effect sizes from dataset 2 and 3. The horizontal lines in (a) represent the medium effect size (0.50) for Cohen’s d and the lines in (b) represent the small effect size for Omega squared (0.01).

We postulated that applying a *p* value threshold to individual voxels may improve the sensitivities to group differences, because the selected ‘activated’ voxels will better represent voxels that show group differences within an ROI. We compared the sensitivities of ROI summary measures 1, 2, 3, 4, 7, and 8 with or without applying *p* threshold to individual voxels (red vs. black bars on Figs 1, 2 and 4). Here we chose a liberal threshold of *p* = 0.05. After applying the *p* threshold, for the two-sample datasets (Fig 4A), the effect sizes for mean (measure 1A, 0.38 vs. 1B, 0.44), median (measure 2A, 0.25 vs. 2B, 0.40), and first eigenimage weighted mean (measure 3A, 0.23 vs. 3B, 0.44) increased for the anatomical ROI; whereas there was no change for other measures. Similarly, for the three-group comparisons (dataset 2 and 3, see Fig 4B), compared to other summary measures, a bigger increase in the effect size was found for measures 1, 2, and 3 for all ROIs (10-mm spherical ROI, measure 1A, 0.012 vs. 1B, 0.021; measure 2A, 0 vs. 2B, 0.017; measure 3A, 0 vs. 3B, 0.021; Anatomical ROI, measure 1A, 0 vs. 1B, 0.013; measure 2A, 0 vs. 2B, 0.004; measure 3A, 0.001 vs. 3B, 0.016; Task activated ROI, measure 1A, 0 vs. 1B, 0.006; measure 3A, 0.003 vs. 3B, 0.011).

The green lines in Fig 3 depict the magnitude and standard errors of measure 1B, ROI mean of all voxels above *p* threshold, and the red lines represent measure 1A, ROI mean without *p* threshold. In general, the ROI mean increased after applying the *p* threshold. For all ROIs in subset 2 of dataset 1, the group difference became significant for 1B. But this did not generalize to other datasets (Fig 2).

In summary, the mean, median, and eigenimage weighted mean measures were greatly affected by the inactive or deactivated voxels in an ROI. By applying a threshold and selecting only the top activated voxels, the effect sizes significantly improved. This finding is consistent with Mitsis et al. [20], who observed that selecting the top fraction voxels of an ROI can increase signal-to-noise ratio and consequently the sensitivity. In comparison, other measures already selected the voxels that are around the peak (sphere, peak-correlated voxels) or above certain threshold (percentile). Thus the effect sizes did not improve much by applying an additional *p*-value threshold.

#### Replicability between the two NBack subsets

We split the first dataset into two matched subsets in order to test the replicability across the ten different ROI single-value summary measures for the three types of ROIs. The results are depicted in Fig 1.

For the 10 mm spherical ROI (Fig 1A), all measures except for the mean, median, and the peak-cluster extent measures, showed consistency across the two subsets. For the bigger anatomical (Fig 1B) and task-activated ROIs (Fig 1C), the top percentile (measure 4), top N voxel (measure 6), sphere (measure 7), and peak-correlated voxels (measure 8) generated significant group difference results (*p* < 0.05) consistently across the two split datasets. As shown in Fig 3A, the group difference magnitude of the peak measure (measure 5) (anatomical ROI, 0.21; task-activated ROI, 0.22) was greater than measure 8 (anatomical ROI, 0.15; task-activated ROI, 0.14). However, measure 8 has much reduced between-subject variance (anatomical ROI, 0.25; task-activated ROI, 0.24) relative to measure 5 (anatomical ROI, 0.56; task-activated ROI, 0.55). In comparison, the ROI mean measure (measure 1) has both smaller group difference magnitude (anatomical ROI, 0.03; task-activated ROI, 0.05) and variance (anatomical ROI, 0.13; task-activated ROI, 0.16). This is the reason why the peak-correlated voxels measure, as well as other measures to select multiple voxels surrounding the peak, could be more sensitive to group difference than the peak and the mean measures for the anatomical and task-activated ROIs. This brings up a question when only a subset of voxels within an ROI are chosen to create the summary measure, whether the results can be extended to the whole ROI. We argue that these selected voxels are around the contrast’s peak, which has been used by many researchers to characterize the activation of the whole ROI. The mean of the selected subset of voxels (including the peak) represents the most highly activated region in each individual’s ROI and can be deemed as a valid measure.

To further examine the replicability of the ROI summary measures, using an automated script we randomly selected 50 PTs and 50 matching NCs from the first dataset. This was done 1000 times. For each sample we calculated the Cohen’s *d* and group *t* test *p* values for each ROI measure. S3A Fig shows the averaged Cohen’s *d* of the 1000 samples for each ROI measure, and S3B Fig shows the percentage of total number of samples (out of the 1000 sampled data) with *t* test *p* < 0.05 and *p* < 0.01, respectively, for each ROI measure. The patterns shown in Fig 1 and S3A Fig are almost identical.

#### Replicability across the NBack datasets

We further compared the replicability of these ROI single-value summary measures by using two more NBack datasets: the sibling dataset and the COMT dataset. Both datasets include three groups, and we used one-way ANOVAs to test for an effect (PTs>SIBs>NCs) in the sibling dataset, and the COMT Val/Met polymorphism dosage effect (Val/Val>Val/Met>Met/Met) in the COMT dataset, respectively (Fig 2A and 2B). We noticed that the significant levels and the effect sizes were smaller relative to the split dataset, where a two-group comparison was conducted. A consistent finding is that the measure of peak-correlated voxels (measure 8) always stood out to be significant for the group comparisons, even though the effect size is in the range between the small (0.01) and medium (0.59) of Omega Squared [38]. In addition, the top percentile voxels measure (measure 4) also detected consistent significant group effects (*p* < 0.05) for the spherical ROI and the anatomical ROI.

#### Replicability across all the datasets

We averaged the Flanker NoGo task NCs vs. PTs group comparison results with the ones generated by the split dataset. Overall the patterns replicated what we observed in dataset 1. For the 10 mm spherical ROI, all measures except for the peak-cluster extent measure generated medium level effect sizes; whereas for anatomical and task-activated cluster, the percentile (measure 4), top N contiguous voxels (measure 6), sphere surrounding the peak (measure 7), and peak-correlated voxels (measure 8) consistently generated medium effect sizes.

### Parameter Comparisons

As stated in the Methods section, we used three different parameters for voxel selections for measure 4 (percentile = top 25%, 10%, and 5%), measure 6 (N = 10, 20, and 50 voxels), measure 7 (radius = 6, 7.5, and 10 mm), and measure 8 (correlation coefficient r = 0.7, 0.8, and 0.9). No significant difference in effect size was observed across these different approaches (S4 Fig).

### Between-Group Small ROI Localization Differences

Some of the measures we are proposing here selected only a smaller subset of voxels out of a large ROI (i.e., measures 4, 5, 6, 7, 8, 9, and 10). Thus, the localization of the smaller cluster should be taken into consideration when comparing the extracted ROI summaries across groups. If the localizations of the selected cluster of voxels are different across groups, then the group difference in magnitude of ROI single value summaries would be confounded by the clusters’ locations. Since most of the ROI summary measures were related to selecting smaller clusters around the peak voxel of the larger ROI, the peak coordinates were used to approximately represent the smaller clusters’ center for each individual contrast map.

As described in the Method section, we used a permutation test to compare the peak localization between groups. S5 Table lists the summary of the permutation results and the percentile of the actual group centroids distance in the permutation distribution for each dataset used in the study. It shows that there is no significant difference between groups in the peak location. Therefore, the group differences we observed in the ROI summary measures were not confounded by ROI localization differences between groups.

### Future directions

In this study, the ROI summary measures were based on image data that were spatially normalized and smoothed, and the ROIs were defined from atlases. The ROI analysis may be more precise if the ROIs are defined based on individual anatomy [20, 23]. Going forward, we plan to examine how ROI summary measures can be improved for the unnormalized and unsmoothed fMRI image data by defining the ROIs in native space using anatomical landmarks.

In this project we only employed three simple ROI definition strategies. More advanced methodologies based on multivariate pattern analysis, such as ICA and multi-voxel pattern analysis (MVPA), also can be used to define ROIs. Further studies should be conducted to see how these new ROI definition strategies would interact with different ROI summary measures.

## Conclusions

This study attempted to provide guidance in defining an optimal single-value summary measure that can be reliably used in genome-wide Imaging Genetics studies. Overall, we found that four ROI single-value summary measures were reliably consistent across datasets to show significant group differences and produce relatively higher effect sizes: 1) top percentile (measure 4), 2) the top N contiguous voxels surrounding the peak voxel (measure 6); 3) a sphere surrounding the peak voxel (measure 7); and 4) peak-correlated voxels (measure 8).

In terms of statistical power, the ROI approach is equivalent or better than the voxel-wise analysis approach in detecting significant group differences. Different ROI summary measures are more similar when using the smaller, focal spherical ROIs. The drawback is that this type of ROI requires a strong *a priori* hypothesis to pinpoint the center of the sphere and most of individual subjects’ peak of activation were not within the sphere selected based on *a priori* coordinates from published literature. Applying a *p* threshold can help in selecting activated voxels within an ROI, especially for the mean, median, and eigenimage weighted mean measures.

## Supporting Information

S1 Table Demographic and performance data of the NBack task split dataset. (DOC)

S2 Table Demographic and performance data of the NBack task sibling dataset. (DOC)

S3 Table Demographic and performance data of the NBack task COMT dataset. (DOC)

S4 Table Demographic and performance data of the Flanker task dataset. (DOC)

S5 Table Summary of the between-group ROI peak location permutation test for each dataset. (DOC)

S1 Fig ROIs used in datasets 1–4. a)10-mm ROI in BA9 used in Dataset 1 and 2; b) The right dorsal lateral prefrontal (DLPFC) anatomical ROI used in Dataset 1, 2 and 3; c) Task-activated cluster in right DLPFC used in Dataset 1, 2 and 3; d) The 10-mm spherical ROI in BA46 used in Dataset 3; e) The 10-mm spherical ROI in dorsal anterior cingulate used in Dataset 4; f) The dorsal anterior cingulate anatomical ROI used in Dataset 4; g) The task-activated cluster in dorsal anterior cingulate anatomical ROI used in Dataset 4.
(TIF)

S2 Fig **Group mean plots for datasets 3 (panel a) and 4 (panel b).** Selected ROI summary measures (peak, mean, mean of voxels above *p* = 0.05 threshold, and peak-correlated voxels) were used to extract single-value summaries from three types of ROIs in right DLPFC. * *p* < 0.05; ** *p* < 0.01. Error bars represent standard errors.
(TIF)

S3 Fig Using the 1000 random samples from the NBack dataset to evaluate the replicability of ROI summary measures. a) Averaged Cohen’s *d* of the 1000 samples for each ROI measure; b) The percentage of total number of samples out of 1000 samples with t test *p* < 0.05 and *p* < 0.01, respectively, for each ROI measure.
(TIF)

S4 Fig Comparisons of effect size for different parameters used in measure 4 (percentile = top 25%, 10%, and 5%), measure 6 (top N = 10, 20, and 50 voxels), measure 7 (sphere radius = 6, 7.5, and 10 mm), and measure 8 (correlation coefficient r = 0.7, 0.8, and 0.9). Panel a shows the combined the results of the two-sample *t* tests by averaging the effect sizes from dataset 1 and 4 together; and panel b shows the combined the one-way ANOVA results by averaging the effect sizes from dataset 2 and 3.
(TIF)

S1 Data Statistics for the four datasets. The statistics include: N, mean, standard deviation, P values, effect size, and power.
(ZIP)

S2 Data The extracted ROI summary values from three types of ROIs for each subject in all datasets. (ZIP)
