# Supplementary material for: Seeking Optimal Region-Of-Interest (ROI) Single-Value Summary Measures for fMRI Studies in Imaging Genetics
Source: PLoS One. 2016 Mar 14;11(3):e0151391. doi: 10.1371/journal.pone.0151391 (PMC4790904; doi:10.1371/journal.pone.0151391)
Supplement: S1 Table — (DOC) [file pone.0151391.s001.doc]

**S1 Table. Demographic and performance data of the NBack task split data sample**

|  | **Subset 1** | | **Subset 2** | | **P Values** | | | |
| --- | --- | --- | --- | --- | --- | --- | --- | --- |
| **Characteristics** | Normal Controls (NC1) | Patients (PT1) | Normal Controls (NC2) | Patients (PT2) | NC1 vs. PT1 | NC2 vs. PT2 | NC1 vs. NC2 | PT1 vs. PT2 |
| **N (total = 200)** | 50 | 50 | 50 | 50 |  |  |  |  |
| **Males/Females** | 36/14 | 36/14 | 36/14 | 36/14 | 1 | 1 | 1 | 1 |
| **Age, mean (SD)** | 33 (10.11) | 33.2 (9.5) | 34.1 (9.7) | 32.2 (9.7) | 0.93 | 0.35 | 0.57 | 0.6 |
| **WRAT, mean (SD)** | 101 (6) | 101 (8.9) | 100 (6.4) | 99.4 (9.6) | 0.97 | 0.68 | 0.45 | 0.41 |
| **Handedness, mean (SD)** | 93.8 (9.5) | 93.7 (8.8) | 95 (8.3) | 95.5 (7.4) | 0.95 | 0.72 | 0.49 | 0.27 |
| **Percent correct answers during**  **2-back, mean (SD)** | 74.1 (16) | 75.6 (17) | 73.6 (20.2) | 72.6 (19.6) | 0.64 | 0.81 | 0.9 | 0.42 |
| **Temporal Signal to Noise Ratio, mean (SD)** | 212.6 (41.3) | 205 (45.7) | 210.8 (35.4) | 204.3 (39.1) | 0.38 | 0.39 | 0.81 | 0.94 |

* SD= standard deviation
