# Supplementary material for: Seeking Optimal Region-Of-Interest (ROI) Single-Value Summary Measures for fMRI Studies in Imaging Genetics
Source: PLoS One. 2016 Mar 14;11(3):e0151391. doi: 10.1371/journal.pone.0151391 (PMC4790904; doi:10.1371/journal.pone.0151391)
Supplement: S2 Table — (DOC) [file pone.0151391.s002.doc]

**S2 Table. Demographic and performance data of the NBack task three-group data sample**

| **Characteristics** | **Normal Controls (NC)** | **Siblings (SIB)** | **Patients (PT)** | **P Values** |
| --- | --- | --- | --- | --- |
| **N (total = 129)** | 43 | 43 | 43 |  |
| **Males/Females** | 33/10 | 33/10 | 33/10 | 1 |
| **Age, mean (SD)** | 31.8 (8.8) | 32.6 (9.6) | 32.1(9.9) | 0.92 |
| **WRAT, mean (SD)** | 106 (8) | 106 (9) | 106.1 (9.9) | 1 |
| **Handedness, mean (SD)** | 94.1 (9) | 96 (9.1) | 96.2 (7.9) | 0.54 |
| **Percent correct answers during**  **2-back, mean (SD)** | 86.4 (10) | 86.2 (10) | 84.6 (9) | 0.62 |
| **Temporal Signal to Noise Ratio, mean (SD)** | 216.5 (37.7) | 211.4 (28.9) | 205 (46.3) | 0.37 |

* SD= standard deviation
