# Supplementary material for: Seeking Optimal Region-Of-Interest (ROI) Single-Value Summary Measures for fMRI Studies in Imaging Genetics
Source: PLoS One. 2016 Mar 14;11(3):e0151391. doi: 10.1371/journal.pone.0151391 (PMC4790904; doi:10.1371/journal.pone.0151391)
Supplement: S3 Table — (DOC) [file pone.0151391.s003.doc]

**S3 Table. Demographic and performance data of the NBack task COMT data sample**

| **Characteristics** | **Val/Val** | **Val/Met** | **Met/Met** | **P Values** |
| --- | --- | --- | --- | --- |
| **N (total = 216)** | 43 | 106 | 67 |  |
| **Males/Females** | 18/25 | 49/57 | 30/37 | 0.89 |
| **Age, mean (SD)** | 30.9 (9.3) | 30 (8) | 30 (8.4) | 0.83 |
| **WRAT, mean (SD)** | 109 (10.6) | 109.1 (6.9) | 108.5 (8.3) | 0.9 |
| **Handedness, mean (SD)** | 94.4 (8.5) | 93 (9) | 93.1 (9.6) | 0.68 |
| **Percent correct answers during**  **2-back, mean (SD)** | 88.5 (10.2) | 89.3 (8.7) | 90.7 (8.5) | 0.42 |
| **Temporal Signal to Noise Ratio, mean (SD)** | 214 (35.2) | 216.6 (31.2) | 215.3 (39.2) | 0.91 |

* SD= standard deviation
