# Supplementary material for: Seeking Optimal Region-Of-Interest (ROI) Single-Value Summary Measures for fMRI Studies in Imaging Genetics
Source: PLoS One. 2016 Mar 14;11(3):e0151391. doi: 10.1371/journal.pone.0151391 (PMC4790904; doi:10.1371/journal.pone.0151391)
Supplement: S4 Table — (DOC) [file pone.0151391.s004.doc]

**S4 Table. Demographic and performance data of the Flanker task data sample**

| **Characteristics** | **Normal Controls (NC)** | **Patients (PT)** | **P Values** |
| --- | --- | --- | --- |
| **N (total = 56)** | 28 | 28 |  |
| **Males/Females** | 22/6 | 22/6 |  |
| **Age, mean (SD)** | 33 (9.9) | 32.9(9.6) | 0.98 |
| **WRAT, mean (SD)** | 104.4 (7.7) | 105.2 (8.2) | 0.69 |
| **Handedness, mean (SD)** | 93.2 (9.8) | 95.9 (9.5) | 0.31 |
| **Percent correct answers for NoGo trials, mean (SD)** | 92.4 (5.7) | 92 (5.4) | 0.77 |
| **Signal to Noise Ratio, mean (SD)** | 184.2 (43.7) | 188 (38.2) | 0.73 |

* SD= standard deviation
