# Supplementary material for: Seeking Optimal Region-Of-Interest (ROI) Single-Value Summary Measures for fMRI Studies in Imaging Genetics
Source: PLoS One. 2016 Mar 14;11(3):e0151391. doi: 10.1371/journal.pone.0151391 (PMC4790904; doi:10.1371/journal.pone.0151391)
Supplement: S5 Table — (DOC) [file pone.0151391.s005.doc]

**S5 Table. Summary of the between-group ROI peak location permutation test for each dataset**

| **Dataset** | **Actual group centroids distance (mm)** | **Average of random distance (mm) from the permutation test** | **95% percentile distance (mm) in the permutation distribution** | **Percentile of the actual group centroid distance in the permutation distribution** |
| --- | --- | --- | --- | --- |
| **NBack subset 1** | 4.42 | 3.92 | 7.51 | 64.3 % |
| **NBack subset 2** | 3.13 | 3.96 | 7.33 | 36.86 % |
| **NBack three group** | 4.73 | 6.91 | 15.16 | 34.92 % |
| **NBack COMT** | 5.07 | 5.65 | 9.43 | 43.66 % |
| **Flanker NOGO** | 5.50 | 4.61 | 7.68 | 73.65 % |
