# Supplementary figures and images for: Seeking Optimal Region-Of-Interest (ROI) Single-Value Summary Measures for fMRI Studies in Imaging Genetics
Source: PLoS One. 2016 Mar 14;11(3):e0151391. doi: 10.1371/journal.pone.0151391 (PMC4790904; doi:10.1371/journal.pone.0151391)

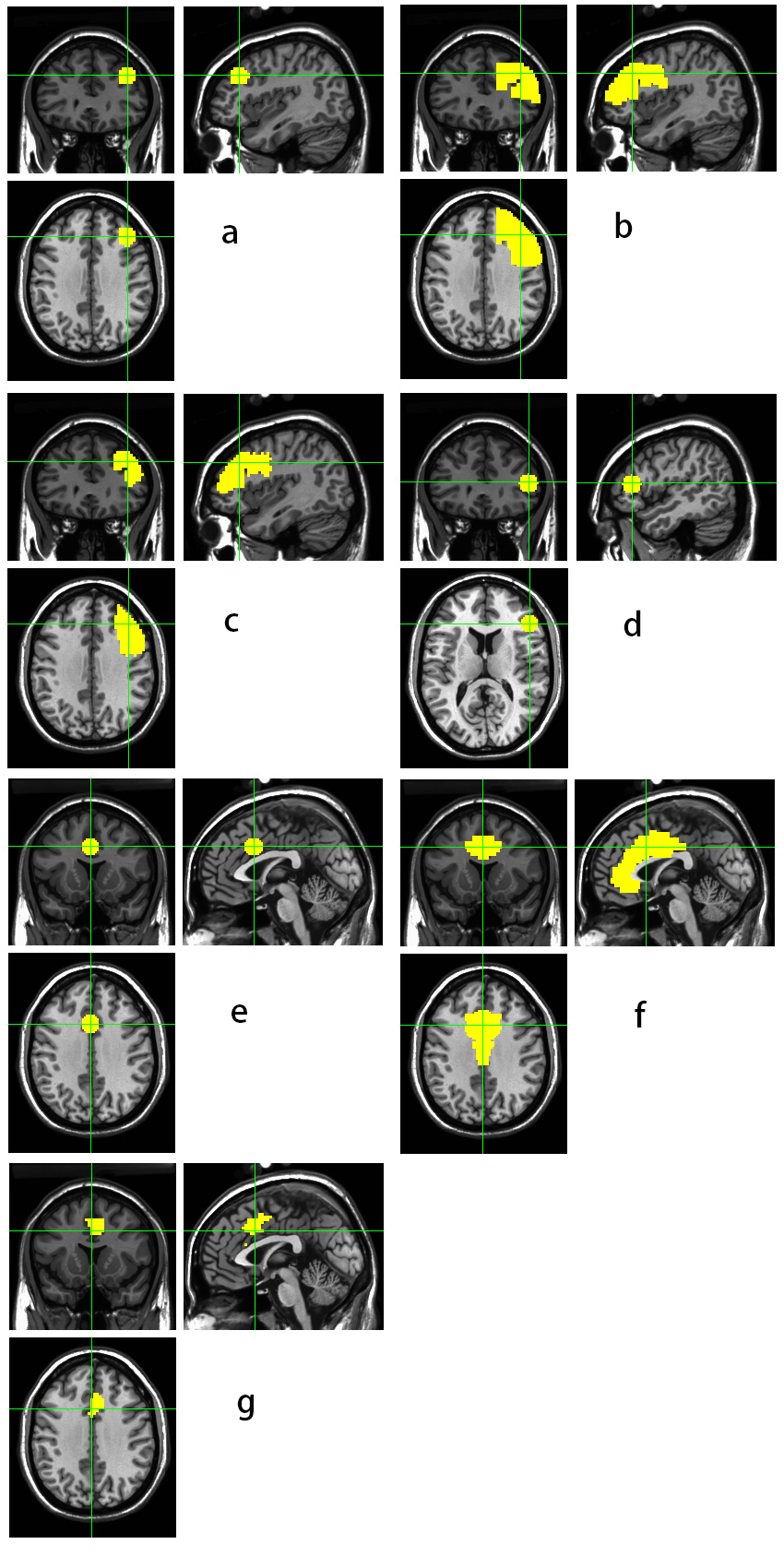

Supplement: S1 Fig — a)10-mm ROI in BA9 used in Dataset 1 and 2; b) The right dorsal lateral prefrontal (DLPFC) anatomical ROI used in Dataset 1, 2 and 3; c) Task-activated cluster in right DLPFC used in Dataset 1, 2 and 3; d) The 10-mm spherical ROI in BA46 used in Dataset 3; e) The 10-mm spherical ROI in dorsal anterior cingulate used in Dataset 4; f) The dorsal anterior cingulate anatomical ROI used in Dataset 4; g) The task-activated cluster in dorsal anterior cingulate anatomical ROI used in Dataset 4. (TIF) [file pone.0151391.s006.tif]

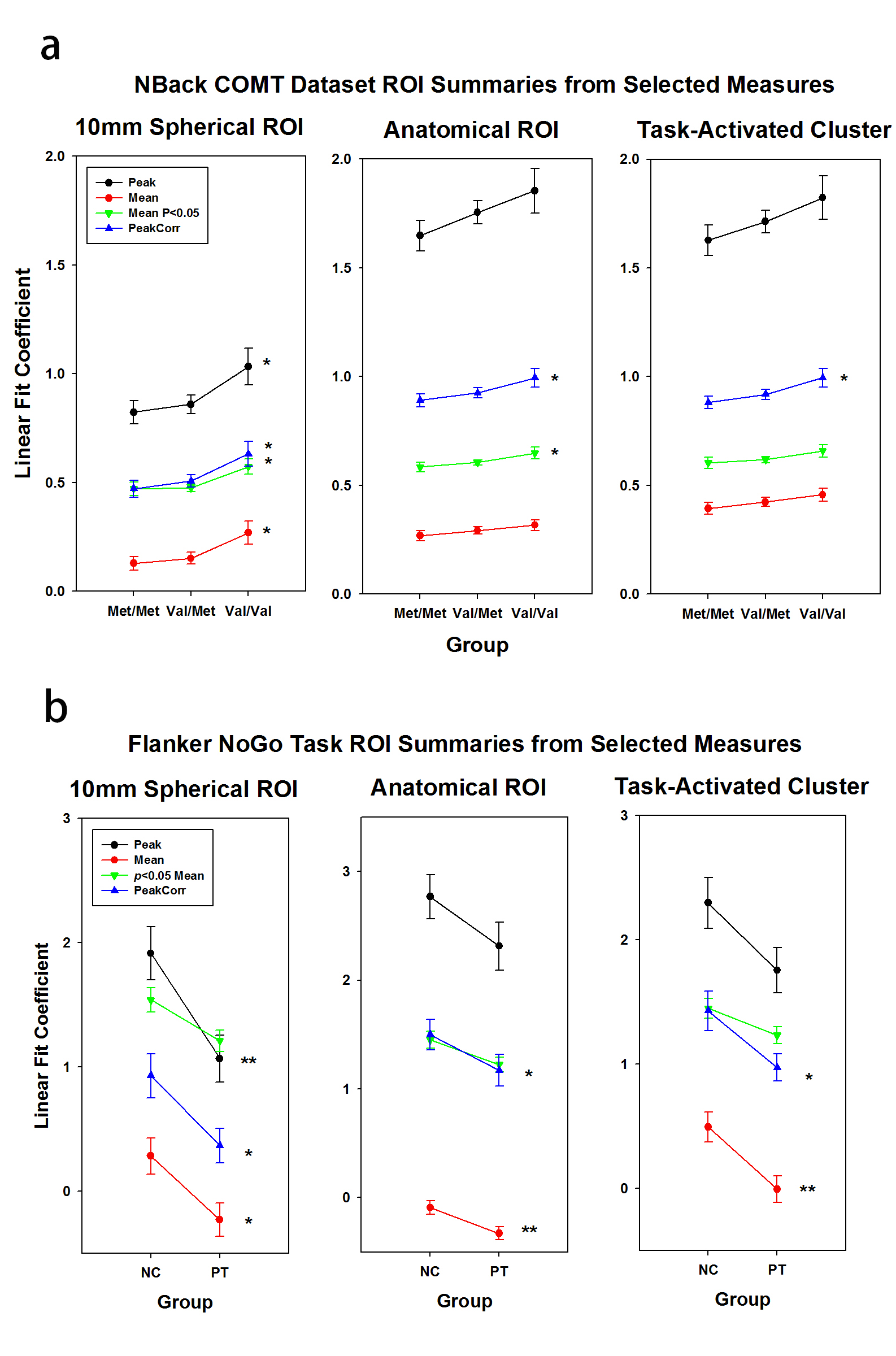

Supplement: S2 Fig — Group mean plots for datasets 3 (panel a) and 4 (panel b). Selected ROI summary measures (peak, mean, mean of voxels above p = 0.05 threshold, and peak-correlated voxels) were used to extract single-value summaries from three types of ROIs in right DLPFC. * p < 0.05; ** p < 0.01. Error bars represent standard errors. (TIF) [file pone.0151391.s007.tif]

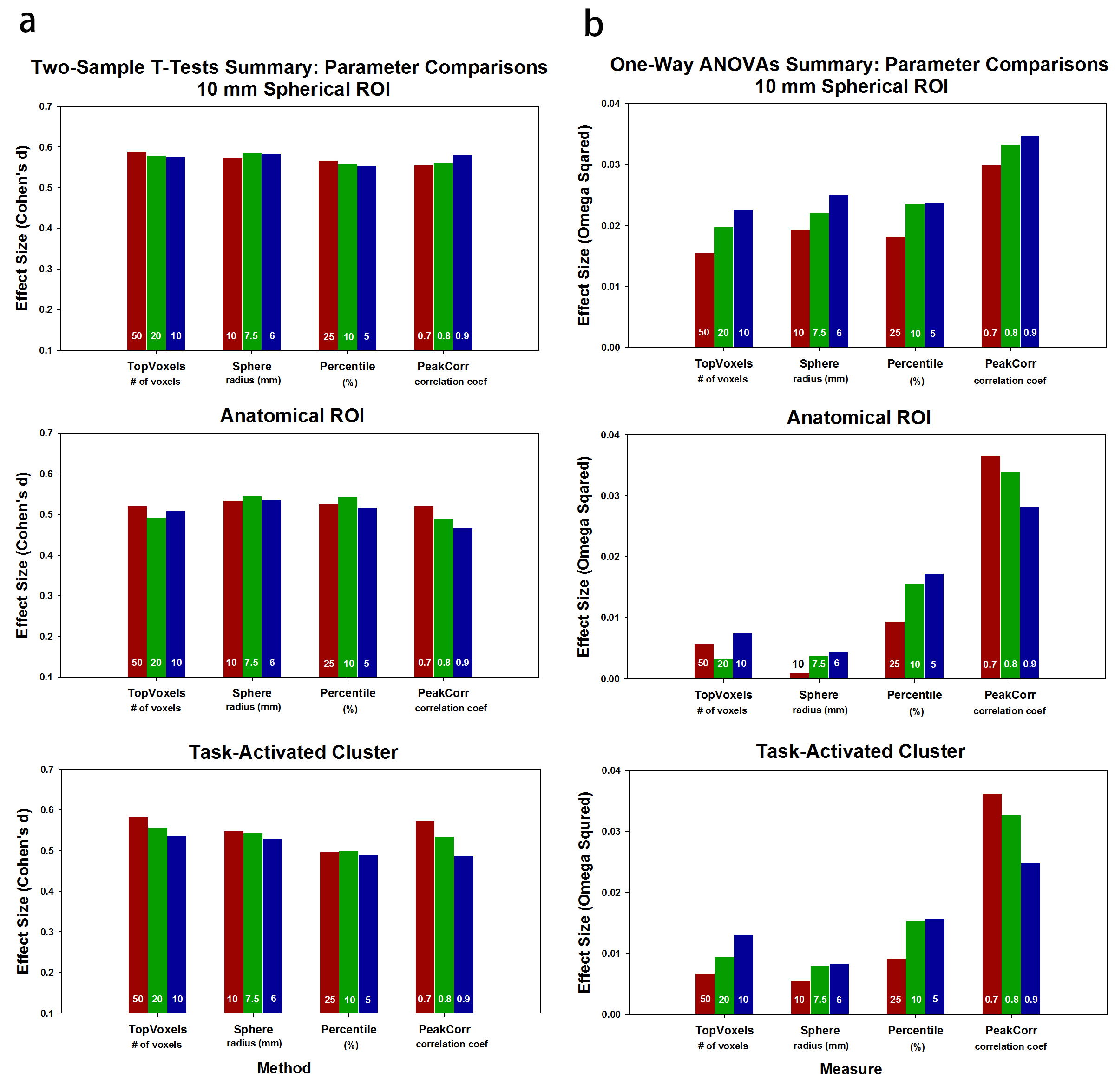

Supplement: S4 Fig — Panel a shows the combined the results of the two-sample t tests by averaging the effect sizes from dataset 1 and 4 together; and panel b shows the combined the one-way ANOVA results by averaging the effect sizes from dataset 2 and 3. (TIF) [file pone.0151391.s009.tif]
